# Influence of Obesity Parameters on Different Regional Patterns of Lymph Node Metastasis in Papillary Thyroid Cancer

**DOI:** 10.1155/2022/3797955

**Published:** 2022-11-07

**Authors:** Wan-Xiao Wu, Jia-Wei Feng, Jing Ye, Gao-Feng Qi, Li-Zhao Hong, Jun Hu, Sheng-Yong Liu, Yong Jiang, Zhen Qu

**Affiliations:** The Third Affiliated Hospital of Soochow University, Changzhou First People's Hospital, Changzhou, Jiangsu, China

## Abstract

**Objective:**

Obesity increases risk of thyroid cancer. However, the association between obesity and the progression of papillary thyroid cancer (PTC) remains controversial. This retrospective study aimed to explore the relationship between obesity and regional patterns of lymph node metastasis (LNM) in PTC.

**Methods:**

We retrospectively reviewed data from 1015 patients with PTC. We calculated obese parameters, such as body mass index (BMI), body fat percentage (BFP), and body surface area (BSA). Logistic regression models were used to assess associations between obese parameters and the rate of lymph node metastasis (LNM), number of LNM, pattern of LNM, and lymph node ratio (LNR).

**Results:**

Higher BMI was not associated with different regional patterns of LNM in PTC. In men with PTC, high BFP was an independent predictor of total LNM, central lymph node metastasis (CLNM), total lateral lymph node metastasis (LLNM), multiple lateral lymph node metastasis, and simultaneous metastasis in lateral compartment. In addition, male patients with high BFP had higher central LNR and higher number of CLNM. For women, high BSA was an independent predictor of LLNM and level IV metastasis. Female patients with high BSA had higher number of CLNM.

**Conclusion:**

BFP and BSA, possibly influenced by gender, were positively associated with the number and risk of LNM in different regions of PTC patients. However, BMI was not the predictor for aggressiveness of PTC in terms of LNM. Clinical decision-making for regional LNM in PTC patients should consider the factor of obesity.

## 1. Introduction

Excessive accumulation of body fat can lead to obesity. In recent decades, the incidence of obesity has risen dramatically and has become a major global public health problem [1]. Cancer and obesity are closely related, especially for thyroid disease, previous studies have found a positive relationship between obesity and thyroid cancer [1–3]. A recent systematic review reported that obese people have a 55% increased risk of developing thyroid cancer compared with lean people [1]. Schmid et al. [4] reported that obesity only increased the risk of papillary thyroid cancer (PTC), follicular thyroid cancer, and anaplastic thyroid cancer, indicating a potential dependence on tumor type and histological specificity. In addition to morbidity, some authors have noted that obesity is associated with aggressive cancer histopathological features and adverse outcomes, especially in breast cancer and prostate cancer [5]. However, whether obesity may contribute to aggressive histopathological features of thyroid cancer remains controversial.

Obesity is usually indicated by a higher body mass index (BMI). There is a significant association between increased BMI and increased incidence of PTC [6]. However, little is known about the association between BMI and clinicopathological features or outcomes of PTC. Paes et al. [7] showed that higher BMI was not associated with more aggressive tumor characteristics of PTC, such as the absence of lymph node metastasis (LNM). In contrast, Li et al. [8] demonstrated that higher BMI was associated with poor prognosis in PTC patients. This paradoxical result may be due to limitations of BMI, such as the inability to distinguish between fat mass and lean mass [9], and the failure to take into account the differences in body composition related to age, gender, etc., [10]. As we all know, obesity can be quantified not only by BMI, but also by body fat percentage (BFP) and body surface area (BSA), which can be obtained by simple measurement and calculation. According to the clinical definition of obesity, its assessment should preferably be based on percent fat content. Hence, some investigators believe that BFP rather than BMI is a more effective indicator of actual obesity [11]. In addition, BSA is positively proportional to basal metabolism, reflecting the ratio of body fat to nonfat components in individuals with the same BMI [12].

The status of lymph nodes determines the prognosis of patients with PTC [13–15]. Hence, lymph nodes status can influence clinical decisions. The association between obesity and the status of lymph nodes in PTC has not been thoroughly investigated. Therefore, we assessed the relationship between obesity indicators (BMI, BFP, and BSA) and patterns of lymph node involvement in different regions through a retrospective analysis of large-scale data, focusing on the incidence of LNM, number of LNM, pattern of LNM, and lymph node ratio (LNR).

## 2. Materials and Methods

### 2.1. Study Design

**Figure 1:**
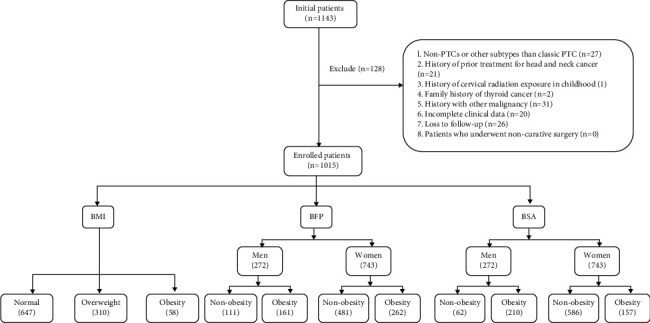
Flowchart of the patients enrolled in this study.

Our study was a retrospective cohort study of patients with PTC. The Institutional Review Board of CHangzhou First People's Hospital approved this retrospective study. The approval number of Institutional Review Board was (2022) Teach No. 024. The need for informed consent was waived due to the retrospective nature of this study. We retrospectively reviewed the medical records of 1143 patients with pathologically proven PTC who underwent primary surgical treatment at our institution between March 2019 and October 2021. Patients were excluded for any of the following factors: nonPTCs or other subtypes than classic PTC, history of prior treatment for head and neck cancer, history of cervical radiation exposure in childhood, family history of thyroid cancer, history with other malignancy, incomplete clinical data, loss to follow-up, and patients who underwent noncurative surgery (residual tumor or lymph node detected within 6 months of initial surgery). A total of 1015 patients were eventually enrolled in this study. The recruitment pathway for patients in this study was displayed in Figure 1.

### 2.2. Surgical Strategy

Fine needle aspiration cytology (FNAC) was conducted to confirm the histopathologic diagnosis preoperatively for suspicious thyroid nodules. For cervical lymph nodes were considered suspicious by ultrasound, FNAC was used to confirm the diagnosis preoperatively. All patients with PTC routinely underwent prophylactic central neck dissection (CND) according to the Chinese guidelines for diagnosis and treatment of differentiated thyroid carcinoma. Ipsilateral CND included the removal of prelaryngeal, pretracheal, and ipsilateral paratracheal lymph nodes, whereas bilateral CND included the removal of prelaryngeal, pretracheal, and bilateral paratracheal lymph nodes [16]. Prophylactic modified radical neck dissection (MRND) was not recommended. Ipsilateral therapeutic MRND was performed for patients with LLNM confirmed by preoperative FNAC or intraoperative frozen pathological examination. MRND referred to the removal of the lateral lymph nodes, including level II to V, while preserving one or more nonlymphatic structures, such as the spinal accessory nerve, internal jugular vein, or sternocleidomastoid muscle. Unless indicated, level I dissection was not routinely performed.

### 2.3. Measurement of Anthropometric Parameters

The demographic and clinical data, including height and weight, were recorded on the first admission. BMI (kg/m^2^) was defined as weight (kg) divided by height (m) squared. According to the World Health Organization-BMI standard, enrolled PTC patients were divided into normal (BMI < 25 kg/m^2^), overweight (25 ≤ BMI < 30 kg/m^2^), and obese (BMI ≥ 30 kg/m^2^) group.

BFP (%) = (1.20 *∗* BMI) + (0.23 *∗* Age) − (10.8 *∗* Sex) − 5.4, where age is in years and sex is set to 0 for women and 1 for men [17]. The American Council on Exercise defines obesity as >25% BFP in men and >31% BFP in women [18]. Hence, enrolled patients were grouped into the following four groups by sex: men (nonobesity (BFP ≤ 25%) and obesity (BFP > 25%)), and women (nonobesity (BFP ≤ 31%) and obesity (BFP > 31%)).

BSA, an indicator of metabolic capacity, is less affected by abnormal fat mass than BMI [19]. BSA (m^2^) = 0.007184 *∗* Weight (kg)^0.425^ *∗* Height (cm)^0.725^. Since there is no general consensus on the classification of BSA, we used the Health Statistics Standard (2002) as cut-off values for BSA, i.e., a BSA higher than the mean for men (1.98 m^2^) and women (1.74 m^2^) as obesity [20, 21]. Patients were grouped into the following groups: men (nonobesity (BSA ≤ 1.98 m^2^) and obesity (BSA > 1.98 m^2^)), and women (nonobesity (BSA ≤ 1.74 m^2^) and obesity (BSA > 1.74 m^2^)).

### 2.4. Clinicopathological Characteristics

Two or more experienced pathologists examined the surgical specimens microscopically. We considered a patient to have chronic lymphocytic thyroiditis (CLT) if any of the following conditions were met: (i) elevated levels of thyroid peroxidase antibodies, (ii) the discovery of diffuse heterogeneity on ultrasonography [22]. Extrathyroidal extension (ETE) was defined as the primary tumor extending through the thyroid capsule to perithyroidal soft tissue such as perithyroidal fat, or involving strap muscles, or extending to surrounding structures such as larynx, trachea, esophagus, recurrent laryngeal nerve, subcutaneous soft tissue, skin, internal jugular vein, or carotid artery [23]. Tumor size was determined as the largest diameter of the primary tumor in multifocal tumors [24]. LNR was defined as metastatic lymph nodes divided by the number of dissected lymph nodes [25, 26]. Pattern of LNM included skip metastasis, multiple lateral lymph node metastasis (MLLNM), and simultaneous metastasis in lateral compartment. Skip metastasis was defined as negative CLNM with positive LLNM [27]. MLLNM referred to the number of metastatic lymph nodes in the lateral compartment more than two [28]. In contrast, solitary lateral lymph node metastasis (SLLNM) was defined as patients with only one metastatic lymph nodes in the lateral compartment regardless of CLNM or not [28]. Simultaneous metastasis referred to more than 1-level metastasis in the lateral compartment [29].

### 2.5. Statistical Analysis

Statistical analysis was performed using SPSS v 25.0 software (Chicago, IL, USA). Mean and standard deviation were used to represent continuous variables. Numbers and percentages were used to represent categorical variables. Continuous variables were examined by the Independent *t*-test or one-way analysis of variance (ANOVA). Categorical variables were analyzed by using Pearson's chi-square test or Fisher's exact test. Significant variables in the univariate analysis were included in the multivariate analysis by using the binary logistic regression test. The characteristics of different regional lymph node involvement were regarded as dependent variables, and BMI, BFP, and BSA group were used as a covariate, respectively, and age (per 10 years), glucose, thyroid stimulating hormone (TSH), cholesterol, triglyceride, tumor size, multifocality, ETE, and CLT were used as adjustment variables.

## 3. Results

### 3.1. Baseline Clinicopathological Characteristics of PTC Patients

Our database included 1143 PTC patients, of which 1015 patients were included in the final analysis (Figure 1). As shown in Table 1, the 1015 patients consisted of 743 women (73.2%) and 272 men (26.8%), with mean age of 43.4 ± 12.2 years. The mean BMI was 24.03 ± 3.88. Overweight and obesity accounted for 30.5% and 5.7% of patients, respectively. The mean BFP and BSA were 24.30 ± 10.49 and 1.70 ± 0.18, respectively. BFP and BSA were divided into male and female groups by gender. Obesity accounted for 15.9% and 20.7% of the male patient group by BFP and BSA criteria, respectively. In the female patient group, obesity accounted for 25.8% in BFP and 15.5% in BSA, respectively.

According to postoperative pathology, 593 patients (58.4%) had CLNM, 211 patients (20.8%) had LLNM, and 22 patients (2.2%) had skip metastasis. Of the 211 patients with LLNM, level IV metastasis was the most common (176/1015, 17.3%), followed by level III metastasis (160/1015, 15.8%). The mean number of CLNM was 2.1 ± 2.9. The mean number of lymph node dissected in central compartment was 7.6 ± 5.0 and the mean central LNR was 0.29 ± 0.33. The mean number of LLNM was 5.3 ± 4.0. The mean number of lymph node dissected in lateral compartment was 28.0 ± 10.9 and the mean lateral LNR was 0.05 ± 0.31. Some other detailed clinicopathological features are shown in Table 1.

### 3.2. Impact of BMI on Different Regional Lymph Node Involvement

We first analyzed the impact of BMI on the rate of LNM. As shown in Table 2, no statistical differences were observed in rates of total LNM, CLNM, LLNM, and level II, III, and IV metastasis in normal, overweight, and obese patients. The rate of level V metastasis in overweight patients with PTC was significantly higher than that in normal-weight patients (*P*=0.013). However, binary logistic regression showed that overweight was not anindependent risk factor for level V metastasis (OR = 1.758, 95% CI: 0.957–3.229, *P*=0.069). We then analyzed the effect of BMI on the number of LNM, LNR, and pattern of LNM, and found that BMI was not the risk factor for above variables.

### 3.3. Impact of BFP on Different Regional Lymph Node Involvement in Men

As shown in Table 3, BFPs were divided into male and female groups by gender. Among male patients, the rate of total LNM was significantly higher in obese PTC patients than in normal-weight patients (77.0% vs. 56.8%, *P* < 0.001). Obese patients had significantly higher rates of CLNM and LLNM than normal-weight patients (74.5% vs. 54.1%, *P* < 0.001; 34.8% vs. 18.9%, *P* < 0.001). After adjusting for confounding factors, binary logistic regression showed that BFP in obese men was an independent risk factor for total LNM (OR = 2.553, 95% CI: 1.510–4.317, *P* < 0.001), CLNM (OR = 2.488, 95% CI: 1.487–4.163, *P* < 0.001), and LLNM (OR = 2.286, 95% CI: 1.286–4.063, *P*=0.005). Univariate analysis showed that the rate of level IV metastasis in obese male patients was higher than that in normal male patients, but multivariate analysis showed that obesity was not an independent risk factor for level IV lymph node metastasis.

In obese male patients, the number of CLNM was higher than in normal-weight patients (3.1 vs. 1.9, *P* < 0.001). It was found that BFP in obese men was an independent risk factor for more than two CLNM (OR = 2.034, 95% CI: 1.183–3.497, *P*=0.010).

The central LNR of obese male patients was significantly higher than that of normal-weight patients (0.40 vs. 0.30, *P*=0.025). It was found that BFP in obese men was an independent risk factor for a central LNR greater than 0.09 (OR = 2.442, 95% CI: 1.471–4.054, *P*=0.001).

We finally analyzed the pattern of LNM. MLLNM and simultaneous metastasis in lateral compartment presented the significant association with obesity (all *P* < 0.05). We further conducted a binary logistic regression and found that BFP in obese men remained the independent risk factor for MLLNM (OR = 2.564, 95% CI: 1.387–4.737, *P*=0.003) and simultaneous metastasis in lateral compartment (OR = 2.096, 95% CI: 1.110–3.958, *P*=0.023).

### 3.4. Impact of BFP on Different Regional Lymph Node Involvement in Women

We then analyzed the impact of BFP on different regional lymph node involvement in female patients. As shown in Table 4, there were no statistically significant differences in rates of LNM, number of LNM, LNR, and pattern of LNM between normal and obese female patients.

### 3.5. Impact of BSA on Different Regional Lymph Node Involvement in Men

According to gender, we divided BSAs into male and female groups. After analyzing the relationship between BSA and different regional lymph node involvement in male patients, we found that BSA in obese men was not a risk factor for rates of LNM, number of LNM, LNR, or pattern of LNM. Details are shown in Table 5.

### 3.6. Impact of BSA on Different Regional Lymph Node Involvement in Women

As shown in Table 6, obese female patients had significantly higher rates of total LLNM and level IV metastasis than normal-weight female patients (24.8% vs. 16.2%, *P*=0.013; 22.3% vs. 13.1%, *P*=0.004). In addition, the number of CLNM in obese women with PTC was significantly higher than that in normal-weight women (2.5 vs. 1.8, *P*=0.014). After adjusting for confounding factors, binary logistic regression showed that BSA in obese women was an independent risk factor for total LLNM (OR = 1.708, 95% CI: 1.119–2.609, *P*=0.013), level IV metastasis (OR = 1.869, 95% CI: 1.214–2.962, *P*=0.005), and more than three CLNM (OR = 1.672, 95% CI: 1.149–2.432, *P*=0.007).

## 4. Discussion

Body fat has been identified as a compelling risk factor for several cancers, including PTC [1–3]. One of the hypotheses about the mechanism by which obesity may increase the risk of thyroid cancer is that it is mediated by increases in thyroid volume, thus an increase in the number of thyroid cells. This hypothesis is supported by the fact that thyroid volume increases with height, weight, BMI, and BSA [30]. Unlike previous studies that focused on the association between obesity and PTC behavior [7, 8], our study has unique factors. First, this is a large study of more than 1000 cases, using strict exclusion and inclusion criteria for accurate analysis. In addition to anthropometric parameters of BMI, we also assessed BFP and BSA. Due to sexual dimorphism in obesity, BFP and BSA were analyzed separately for men and women.

We first analyzed the effect of BMI on cervical lymph nodes and found that BMI was not a risk factor for the rate of LNM, number of LNM, pattern of LNM, and LNR. Our results are consistent with previous studies that the increased risk associated with higher BMI did not vary with the presence or absence of adverse cancer histopathological features [31, 32]. Notably, clinical BMI as the sole criterion for assessing obesity has certain limitations. First, BMI does not measure the body fat directly. Second, the composition of body adipose tissue changes with age. Third, the relationship between BMI and body fat differs between men and women. Furthermore, studies even showed that patients assigned to be overweight according to BMI parameters had the same or better health outcomes than patients with normal BMI [33]. These limitations may be important reasons for the above results.

BMI cannot explain the large differences in body composition and fat content between individuals with similar body types, such as adipose tissue having a much lower metabolic rate than fat-free mass. Hence, some other indicators such as BFP and BSA have been proposed as supplements for obesity assessment [11, 12]. As for BFP, it takes into account the effects of age and gender to estimate body fat content. BSA explains differences between muscle and fat better than BMI, especially for individuals with the same BMI. To date, only few studies have examined the association of BSA and BFP with thyroid cancer [34–37], but no studies have investigated the effect of BFP and BSA on PTC behavior. We then analyzed the effect of BFP and BSA on lymph nodes status in both sexes. In men, high BFP was an independent predictor of LNM (including total LNM, CLNM, and total LLNM), MLLNM, and simultaneous metastasis in lateral compartment. In addition, male patients with high BFP had higher central LNR and higher number of CLNM. But in women, there was no significant association between lymph node status and BFP. For women, higher BSA was an independent predictor of LLNM and level IV metastasis. Moreover, female patients with high BSA had higher number of CLNM. However, no association was found between lymph node status and BFP in men. This study demonstrates gender dimorphism in the association between lymph node status and anthropometric parameters of PTC patients. The reason for these differences may be that women have more body fat than men, even with the same relative BMI [38]. Furthermore, unlike men who mainly store fat predominately in the abdomen, women tend to accumulate fat subcutaneously [39]. Therefore, we believe that BFP can be used as a supplementary indicator of obesity to assess the status of cervical lymph nodes in male PTC patients, while BSA can be used in female PTC patients.

According to the molecular mechanism of obesity-tumor association, obesity can promote tumor invasion and metastasis through various obesity-related factors and metabolic pathways [40]. Certain adipokines, such as leptin and adiponectin, have also been implicated as mediators of the effects of obesity on the progression of thyroid cancer. For example, obesity leads to a decrease in adiponectin, which can increase the activity of tumor suppressors such as P53, inhibiting tumor growth and survival [41]. Fan and Li [42] found a strong correlation between leptin and the aggressiveness of thyroid cancer. Hormonal changes, such as TSH and insulin, are also associated with obesity [43]. The growth and differentiation of thyroid cells may be influenced by TSH [44]. About 30% of the rest energy expenditure are regulated by thyroid hormones, which even generated the hypothesis that thyroid hormone substitution with TSH-titration into the lower reference levels may prevent body weight gain. The growth and differentiation of thyroid cells are also influenced by TSH. Previous study found that BSA was independently associated to serum peak TSH concentrations [45, 46]. This is the reason why BSA is proportional to basal metabolism with thyroid cancer.

We believe that the detection of aggressive features of PTC, especially the status of cervical lymph nodes, is crucial in the management of patients with PTC from preoperative examination, surgical approach, to postoperative follow-up. Since the association between BMI and certain histopathological parameters is controversial in PTC patients, we applied other anthropometric factors, namely, BFP and BSA, to study obesity and lymph node status in PTC patients. This is the first analysis demonstrating gender dimorphism in which histopathological parameters of lymph nodes are strongly associated with higher BFP and BSA. These findings may aid clinical decision-making for regional LNM in PTC patients. For example, given the higher risk of LNM, male patients with high BFP or female patients with high BSA should undergo a detailed preoperative examination by an experienced sonographer. Furthermore, given that obese patients may have more positive lymph nodes and that obesity may increase the risk of inadequate lymph node dissection during surgery, surgeons should exercise more caution when performing CND for these patients. The presence of high BFP and BSA should be reported in the specimen's referral as a reminder to pathologists to be more meticulous in the lymph nodes and tumor evaluation. In addition, increased vigilance for occult LLNM may be warranted in women with high BSA or men with high BFP if receiving CND only. These patients should be followed up more closely postoperatively. If suspicious lymph nodes are detected in the lateral compartment after surgery, FNAC should be actively conducted to confirm the histopathological diagnosis, and therapeutic MRND should be performed if necessary.

There were several potential limitations to our study. First, our study was a retrospective study, which is based on single-center data, and tended to have selection biases. Despite of strictly following the inclusion and exclusion criteria, we still could not rule out the possibility of residual confounding variables of measured or unmeasured factors, such as type of diet. Second, our study lacked long-termfollow-up data from patients. Hence, we could not analyze relationship between the risk of obesity and PTC recurrence. Third, while BMI, BFP, and BSA were most commonly used as measures of body mass and fat, other measures such as neck circumference, skin-fold thickness, waist to and hip ratio circumference and regional fat content were not applicable in this study. Fourth, the relationship between weight change and PTC risk has not been uniformly concluded. It has been suggested that changes in obesity over time were unlikely to significantly affect the relationship between obesity and PTC risk [35], while others have shown that weight gain increased the risk of developing TC, and weight loss decreased the risk [47, 48]. Because the measure of obesity was based on records at first admission, we lacked a dynamic measure of changes in obesity. Therefore, the interpretation of our findings is based on the assumption that anthropometric parameters maintain long-term balance and lack dynamic observation. Whether changes in body weight affect the aggressiveness of PTC remains to be further investigated. Finally, the patients enrolled in our study were all Chinese. We will conduct prospective multicenter institutional trials in subsequent studies to obtain more objective conclusions.

In summary, high BMI does not reflect the aggressiveness of PTC in terms of lymph nodes. BFP and BSA can be used as supplementary indicators of obesity to assess the status of cervical lymph nodes in male and female PTC patients, respectively. The possibility of the higher risk of LNM and higher number of CLNM in obese patients should be considered during the decision-making process.

## Figures and Tables

**Table 1:** Baseline clinical characteristics of patients with PTC.

Characteristics	Results
Sex	
Men	272 (26.8%)
Women	743 (73.2%)
Age (mean ± SD, years)	43.4 ± 12.2
≥55	195 (19.2%)
<55	820 (80.8%)
BMI (Mean ± SD, kg/m^2^)	24.03 ± 3.88
Normal	647 (63.7%)
Overweight	310 (30.5%)
Obesity	58 (5.7%)
BFP (Mean ± SD,%)	24.30 ± 10.49
Nonobesity (men)	111 (10.9%)
Obesity (men)	161 (15.9%)
Nonobesity (women)	481 (47.4%)
Obesity (women)	262 (25.8%)
BSA (mean ± SD, m^2^)	1.70 ± 0.18
Nonobesity (men)	62 (6.1%)
Obesity (men)	210 (20.7%)
Nonobesity (women)	586 (57.7%)
Obesity (women)	157 (15.5%)
Maximum tumor size (Mean ± SD, cm)	1.32 ± 1.00
≤1	531 (52.3%)
>1 to ≤2	325 (32.0%)
>2 to ≤4	134 (13.2%)
>4	25 (2.5%)
The number of foci	
1	679 (66.9%)
2	223 (22.0%)
3 or more	113 (11.1%)
BRAF V600E mutation	839 (82.7%)
CLT	272 (26.8%)
ETE	207 (20.4%)
CLNM	593 (58.4%)
LLNM	211 (20.8%)
Level II metastasis	94 (9.3%)
Level III metastasis	160 (15.8%)
Level IV metastasis	176 (17.3%)
Level V metastasis	48 (4.7%)
Skip metastasis	22 (2.2%)
MLLNM	181 (17.8%)
Simultaneous metastasis in LC	162 (16.0%)
No. of removed LNs in CC (mean ± SD)	7.6 ± 5.0
No. of metastatic LNs in CC (mean ± SD)	2.1 ± 2.9
Central LNR (mean ± SD)	0.29 ± 0.33
No. of removed LNs in LC (mean ± SD)	28.0 ± 10.9
No. of metastatic LNs in LC (mean ± SD)	5.3 ± 4.0
Lateral LNR (mean ± SD)	0.05 ± 0.31

PTC, papillary thyroid carcinoma; SD, standard deviation; BMI, body mass index; BFP, body fat percentage; BSA, body surface area; CLT, chronic lymphocytic thyroiditis; ETE, extrathyroidal extension; CLNM, central lymph node metastasis; LLNM, lateral lymph node metastasis; MLLNM, multiple lateral lymph node metastasis; LN, lymph node; CC, central compartment; LC, lateral compartment; and LNR, lymph node ratio.

**Table 2:** Relationship between BMI and different regional lymph node involvement.

	Univariate analysis				Multivariate analysis			
	Normal	Overweight	Obesity	*P* value	Normal	Overweight	Obesity	*P* value
Rate of LNM								
Rate of total LNM	395 (61.1%)	191 (61.6%)	29 (50.0%)	0.233				
Rate of CLNM	383 (59.2%)	183 (59.0%)	27 (46.6%)	0.168				
Rate of total LLNM	134 (20.7%)	67 (21.6%)	10 (17.3%)	0.751				
Rate of level II metastasis	63 (9.7%)	25 (8.1%)	6 (10.3%)	0.676				
Rate of level III metastasis	104 (16.1%)	46 (14.8%)	10 (17.2%)	0.843				
Rate of level IV metastasis	111 (17.2%)	57 (18.4%)	8 (13.8%)	0.683				
Rate of level V metastasis	27 (4.2%)	21 (6.8%)	0 (0.0%)	0.013	Ref	1.758 (0.957–3.229)		0.069

Number of LNM								
Number of CLNM	2.15 (2.95)	2.10 (2.70)	2.05 (3.10)	0.950				
Number of LLNM	4.99 (3.65)	5.64 (4.80)	6.60 (3.27)	0.308				

LNR								
Central LNR	0.30 (0.33)	0.28 (0.32)	0.23 (0.30)	0.324				
Lateral LNR	0.06 (0.37)	0.04 (0.10)	0.04 (0.10)	0.697				

Pattern of LNM								
Skip metastasis	12 (1.9%)	8 (2.6%)	2 (3.4%)	0.628				
MLLNM	116 (17.9%)	57 (18.4%)	8 (13.8%)	0.699				
Simultaneous metastasis in LC	107 (16.5%)	47 (15.2%)	8 (13.8%)	0.567				

The categorical variables were expressed as *n* (%). The continuous variables were expressed as the mean (standard deviations). Multivariate analysis was expressed as adjusted odds ratio (95% CI). BMI, body mass index; OR, odds ratio; LNM, lymph node metastasis; LLNM, lateral lymph node metastasis; CLNM, central lymph node metastasis; MLLNM, multiple lateral lymph node metastasis; LNR, lymph node ratio; and LC, lateral compartment. Age (per 10 years), glucose, thyroid stimulating hormone, cholesterol, triglyceride, tumor size, multifocality, extrathyroidal extension, and chronic lymphocytic thyroiditis as covariates to adjust OR value.

**Table 3:** Relationship between BFP and different regional lymph node involvement in men.

	Univariate analysis			Multivariate analysis		
	Nonobesity (BFP)	Obesity (BFP)	*P* value	Nonobesity (BFP)	Obesity (BFP)	*P* value
Rate of LNM						
Rate of total LNM	63 (56.8%)	124 (77.0%)	<0.001	Ref	2.553 (1.510–4.317)	<0.001
Rate of CLNM	60 (54.1%)	120 (74.5%)	<0.001	Ref	2.488 (1.487–4.163)	<0.001
Rate of total LLNM	21 (18.9%)	56 (34.8%)	0.004	Ref	2.286 (1.286–4.063)	0.005
Rate of level II metastasis	10 (9.0%)	23 (14.3%)	0.190			
Rate of level III metastasis	18 (16.2%)	40 (24.8%)	0.088			
Rate of level IV metastasis	17 (15.3%)	47 (29.2%)	0.008	Ref	1.647 (0.818–3.319)	0.162
Rate of level V metastasis	6 (5.4%)	15 (9.3%)	0.235			

Number of LNM						
Number of CLNM^1^	1.9 (2.6)	3.1 (3.3)	0.001	Ref	2.034 (1.183–3.497)	0.010
Number of LLNM	5.3 (3.8)	5.6 (4.2)	0.764			

LNR						
Central LNR^2^	0.30 (0.35)	0.40 (0.35)	0.025	Ref	2.442 (1.471–4.054)	0.001
Lateral LNR	0.04 (0.11)	0.09 (0.31)	0.052			

Pattern of LNM						
Skip metastasis	3 (2.7%)	4 (2.5%)	0.911			
MLLNM	17 (15.3%)	51 (31.7%)	0.002	Ref	2.564 (1.387–4.737)	0.003
Simultaneous metastasis in LC	16 (14.4%)	42 (26.1%)	0.021	Ref	2.096 (1.110–3.958)	0.023

The categorical variables were expressed as *n* (%). The continuous variables were expressed as the mean (standard deviations). Multivariate analysis was expressed as adjusted odds ratio (95% CI). BFP, body fat percentage; LNM, lymph node metastasis; LLNM, lateral lymph node metastasis; CLNM, central lymph node metastasis; MLLNM, multiple lateral lymph node metastasis; LNR, lymph node ratio; and LC, lateral compartment. Age (per 10 years), glucose, thyroid stimulating hormone, cholesterol, triglyceride, tumor size, multifocality, extrathyroidal extension, and chronic lymphocytic thyroiditis as covariates to adjust OR value. ^1^The cut-off point of number of CLNM is 2 in the multivariate analysis. ^2^The cut-off point of number of Central LNR is 0.09 in the multivariate analysis.

**Table 4:** Relationship between BFP and different regional lymph node involvement in women.

	Univariate analysis		
	Nonobesity (BFP)	Obesity (BFP)	*P* value
Rate of LNM			
Rate of total LNM	283 (58.8%)	145 (55.3%)	0.357
Rate of CLNM	276 (57.4%)	137 (52.3%)	0.182
Rate of total LLNM	91 (18.9%)	43 (16.4%)	0.396
Rate of level II metastasis	42 (8.7%)	19 (7.3%)	0.483
Rate of level III metastasis	74 (15.4%)	28 (10.7%)	0.075
Rate of level IV metastasis	74 (15.4%)	38 (14.5%)	0.749
Rate of level V metastasis	16 (3.3%)	11 (4.2%)	0.544

Number of LNM			
Number of CLNM	1.7 (2.3)	1.9 (2.4)	0.511
Number of LLNM	5.0 (3.6)	5.5 (4.8)	0.527

LNR			
Central LNR	0.23 (0.29)	0.28 (0.32)	0.051
Lateral LNR	0.05 (0.39)	0.03 (0.09)	0.434

Pattern of LNM			
Skip metastasis	7 (1.5%)	8 (3.1%)	0.139
MLLNM	75 (15.6%)	38 (14.5%)	0.693
Simultaneous metastasis in LC	71 (14.8%)	33 (12.6%)	0.416

The categorical variables were expressed as *n* (%). The continuous variables were expressed as the mean (standard deviations). Multivariate analysis was expressed as adjusted odds ratio (95% CI). BFP, body fat percentage; LNM, lymph node metastasis; LLNM, lateral lymph node metastasis; CLNM, central lymph node metastasis; MLLNM, multiple lateral lymph node metastasis; LNR, lymph node ratio; and LC, lateral compartment. Age (per 10 years), glucose, thyroid stimulating hormone, cholesterol, triglyceride, tumor size, multifocality, extrathyroidal extension, and chronic lymphocytic thyroiditis as covariates to adjust OR value.

**Table 5:** Relationship between BSA and different regional lymph node involvement in men.

	Univariate analysis		
	Nonobesity (BSA)	Obesity (BSA)	*P* value
Rate of LNM			
Rate of total LNM	44 (71.0%)	143 (68.1%)	0.668
Rate of CLNM	43 (69.4%)	137 (65.2%)	0.547
Rate of total LLNM	16 (25.8%)	61 (29.0%)	0.619
Rate of level II metastasis	4 (6.5%)	29 (13.8%)	0.119
Rate of level III metastasis	11 (17.7%)	47 (22.4%)	0.433
Rate of level IV metastasis	12 (19.4%)	52 (24.8%)	0.378
Rate of level V metastasis	2 (3.2%)	19 (9.0%)	0.131

Number of LNM			
Number of CLNM	2.3 (2.4)	2.7 (3.3)	0.468
Number of LLNM	5.2 (4.0)	5.5 (4.1)	0.759

LNR			
Central LNR	0.34 (0.31)	0.36 (0.37)	0.684
Lateral LNR	0.04 (0.09)	0.08 (0.28)	0.316

Pattern of LNM			
Skip metastasis	1 (1.6%)	6 (2.9%)	0.930
MLLNM	14 (22.6%)	54 (25.7%)	0.617
Simultaneous metastasis in LC	9 (14.5%)	49 (23.3%)	0.136

The categorical variables were expressed as *n* (%). The continuous variables were expressed as the mean (standard deviations). Multivariate analysis was expressed as adjusted odds ratio (95% CI). BSA, body surface area; LNM, lymph node metastasis; LLNM, lateral lymph node metastasis; CLNM, central lymph node metastasis; MLLNM, multiple lateral lymph node metastasis; LNR, lymph node ratio; and LC, lateral compartment. Age (per 10 years), glucose, thyroid stimulating hormone, cholesterol, triglyceride, tumor size, multifocality, extrathyroidal extension, and chronic lymphocytic thyroiditis as covariates to adjust OR value.

**Table 6:** Relationship between BSA and different regional lymph node involvement in women.

	Univariate analysis			Multivariate analysis		
	Nonobesity (BSA)	Obesity (BSA)	*P* value	Nonobesity (BSA)	Obesity (BSA)	*P* value
Rate of LNM						
Rate of total LNM	331 (56.5%)	97 (61.8%)	0.233			
Rate of CLNM	318 (54.3%)	95 (60.5%)	0.162			
Rate of total LLNM	95 (16.2%)	39 (24.8%)	0.013	Ref	1.708 (1.119–2.609)	0.013
Rate of level II metastasis	44 (7.5%)	17 (10.8%)	0.178			
Rate of level III metastasis	76 (13.0%)	26 (16.6%)	0.246			
Rate of level IV metastasis	77 (13.1%)	35 (22.3%)	0.004	Ref	1.869 (1.214–2.962)	0.005
Rate of level V metastasis	19 (3.2%)	8 (5.1%)	0.270			

Number of LNM						
Number of CLNM^1^	1.8 (2.7)	2.5 (3.1)	0.014	Ref	1.672 (1.149–2.432)	0.007
Number of LLNM	5.1 (3.8)	5.4 (4.7)	0.714			

LNR						
Central LNR	0.26 (0.32)	0.28 (0.31)	0.560			
Lateral LNR	0.05 (0.36)	0.05 (0.11)	0.939			

Pattern of LNM						
Skip metastasis	13 (2.2%)	2 (1.3%)	0.669			
MLLNM	83 (14.2%)	30 (19.1%)	0.125			
Simultaneous metastasis in LC	76 (13.0%)	28 (17.8%)	0.119			

The categorical variables were expressed as *n* (%). The continuous variables were expressed as the mean (standard deviations). Multivariate analysis was expressed as adjusted odds ratio (95% CI). BSA, body surface area; LN, lymph node metastasis; LLNM, lateral lymph node metastasis; CLNM, central lymph node metastasis; MLLNM, multiple lateral lymph node metastasis; LNR, lymph node ratio; and LC, lateral compartment. Age (per 10 years), glucose, thyroid stimulating hormone, cholesterol, triglyceride, tumor size, multifocality, extrathyroidal extension, and chronic lymphocytic thyroiditis as covariates to adjust OR value. ^1^The cut-off point of number of CLNM is 2.5 in the multivariate analysis.

## Data Availability

The data that support the findings of this study are available on request from the corresponding author. The data are not publicly available due to privacy or ethical restrictions.

## References

[1] Harikrishna A., Ishak A., Ellinides A. (2019). The impact of obesity and insulin resistance on thyroid cancer: a systematic review. *Maturitas*.

[2] Schaller S. J., Anstey M., Blobner M. (2016). Early, goal-directed mobilisation in the surgical intensive care unit: a randomised controlled trial. *Lancet*.

[3] Chen W., Zheng R., Baade P. D. (2016). Cancer statistics in China, 2015. *CA: A Cancer Journal for Clinicians*.

[4] Schmid D., Ricci C., Behrens G., Leitzmann M. F. (2015). Adiposity and risk of thyroid cancer: a systematic review and meta-analysis. *Obesity Reviews*.

[5] Lauby-Secretan B., Scoccianti C., Loomis D., Grosse Y., Bianchini F., Straif K. (2016). Body fatness and cancer-viewpoint of the IARC working group. *New England Journal of Medicine*.

[6] Fussey J. M., Beaumont R. N., Wood A. R., Vaidya B., Smith J., Tyrrell J. (2020). Does obesity cause thyroid cancer? a mendelian randomization study. *Journal of Clinical Endocrinology and Metabolism*.

[7] Paes J. E., Hua K., Nagy R., Kloos R. T., Jarjoura D., Ringel M. D. (2010). The relationship between body mass index and thyroid cancer pathology features and outcomes: a clinicopathological cohort study. *Journal of Clinical Endocrinology and Metabolism*.

[8] Li C., Dionigi G., Liang N., Guan H., Sun H. (2021). The relationship between body mass index and different regional patterns of lymph node involvement in papillary thyroid cancers. *Frontiers in Oncology*.

[9] Willett W. C., Dietz W. H., Colditz G. A. (1999). Guidelines for healthy weight. *New England Journal of Medicine*.

[10] Schorr M., Dichtel L. E., Gerweck A. V. (2018). Sex differences in body composition and association with cardiometabolic risk. *Biology of Sex Differences*.

[11] Ho-Pham L. T., Lai T. Q., Nguyen M. T. T., Nguyen T. V. (2015). Relationship between body mass index and percent body fat in vietnamese: implications for the diagnosis of obesity. *PLoS One*.

[12] Roy S. K., Zeb I., Kadakia J., Li D., Budoff M. J. (2012). Body surface area is a predictor of coronary artery calcium, whereas body mass index is not. *Coronary Artery Disease*.

[13] Grogan R. H., Kaplan S. P., Cao H. (2013). A study of recurrence and death from papillary thyroid cancer with 27 years of median follow-up. *Surgery*.

[14] Beasley N. J. P., Lee J., Eski S., Walfish P., Witterick I., Freeman J. L. (2002). Impact of nodal metastases on prognosis in patients with well-differentiated thyroid cancer. *Archives of Otolaryngology-Head and Neck Surgery*.

[15] Ito Y., Kudo T., Kobayashi K., Miya A., Ichihara K., Miyauchi A. (2012). Prognostic factors for recurrence of papillary thyroid carcinoma in the lymph nodes, lung, and bone: analysis of 5, 768 patients with average 10-yearfollow-up. *World Journal of Surgery*.

[16] Carty S. E., Cooper D. S., Doherty G. M. (2009). Consensus statement on the terminology and classification of central neck dissection for thyroid cancer. *Thyroid*.

[17] Deurenberg P., Weststrate J. A., Seidell J. C. (1991). Body mass index as a measure of body fatness: age-andsex-specific prediction formulas. *British Journal of Nutrition*.

[18] Waisbren E., Rosen H., Bader A. M., Lipsitz S. R., Rogers S. O., Eriksson E. (2010). Percent body fat and prediction of surgical site infection. *Journal of the American College of Surgeons*.

[19] Du Bois D., Du Bois E. F. (1989). A formula to estimate the approximate surface area if height and weight be known. *Journal of Nutrition*.

[20] Belvis A. (2004). Health statistics-key data on health 2002 (data 1970–2001) Luxembourg: office for official publications of the European communities, 2002. *Epidemiology Biostats & Public Health*.

[21] Heaf J. G. (2007). The origin of the 1·73-m^2^ body surface area normalization: problems and implications. *Clinical Physiology and Functional Imaging*.

[22] Grani G., Carbotta G., Nesca A. (2015). A comprehensive score to diagnose Hashimoto’s thyroiditis: a proposal. *Endocrine*.

[23] Tuttle R. M., Haugen B., Perrier N. D. (2017). Updated American joint committee on cancer/tumor-node-metastasis staging system for differentiated and anaplastic thyroid cancer (eighth edition): what changed and why?. *Thyroid*.

[24] Feng J. W., Qu Z., Qin A. C., Pan H., Ye J., Jiang Y. (2020). Significance of multifocality in papillary thyroid carcinoma. *European Journal of Surgical Oncology*.

[25] Mansour J., Sagiv D., Alon E., Talmi Y. (2018). Prognostic value of lymph node ratio in metastatic papillary thyroid carcinoma. *Journal of Laryngology & Otology*.

[26] Feng J. W., Ye J., Hong L. Z. (2022). Nomograms for the prediction of lateral lymph node metastasis in papillary thyroid carcinoma: stratification by size. *Frontiers in Oncology*.

[27] Hou J., Zhang Y., Fan Y., Wu B. (2021). Risk factors of skip lateral lymph node metastasis in papillary thyroid carcinoma. *European Archives of Oto-Rhino-Laryngology*.

[28] Yang Q., Chen P., Hu H. Y. (2020). >Preoperative sonographic and clinicopathological predictors for solitary lateral neck node metastasis in papillary thyroid carcinoma: a retrospective study<>. *Cancer Management and Research*.

[29] Li C., Meng Z. Z., Qin J. W., Qiu X. G. (2021). Analysis of risk factors of level V lymphatic metastasis for papillary thyroid carcinoma with pN1b. *JAMA Oncology*.

[30] Boas M., Hegedüs L., Feldt-Rasmussen U., Skakkebaek N. E., Hilsted L., Main K. M. (2009). Association of thyroid gland volume, serum insulin-like growth factor-I, and anthropometric variables in euthyroid prepubertal children. *The Journal of Cinical Endocrinology and Metabolism*.

[31] Rahman S. T., Pandeya N., Neale R. E. (2020). Obesity is associated with BRAF (V600E)-mutated thyroid cancer. *Thyroid*.

[32] Li C. L., Dionigi G., Zhao Y. S., Liang N., Sun H. (2020). Influence of body mass index on the clinicopathological features of 13, 995 papillary thyroid tumors. *Journal of Endocrinological Investigation*.

[33] McAllister E. J., Dhurandhar N. V., Keith S. W. (2009). Ten putative contributors to the obesity epidemic. *Critical Reviews in Food Science and Nutrition*.

[34] Hwang Y., Lee K. E., Park Y. J. (2016). Annual average changes in adult obesity as a risk factor for papillary thyroid cancer: a large-scalecase-control study. *Medicine*.

[35] Xhaard C., de Vathaire F., Cléro E. (2015). Anthropometric risk factors for differentiated thyroid cancer in young men and women from eastern France: a case-control study. *American Journal of Epidemiology*.

[36] Cléro E., Leux C., Brindel P. (2010). Pooled analysis of two case-control studies in New Caledonia and French polynesia of body mass index and differentiated thyroid cancer: the importance of body surface area. *Thyroid*.

[37] Xu L., Port M., Landi S. (2014). Obesity and the risk of papillary thyroid cancer: a pooled analysis of three case-control studies. *Thyroid*.

[38] Andersson D. P., Arner E., Hogling D. E., Ryden M., Arner P. (2017). Abdominal subcutaneous adipose tissue cellularity in men and women. *International Journal of Obesity*.

[39] Palmer B. F., Clegg D. J. (2015). The sexual dimorphism of obesity. *Molecular and Cellular Endocrinology*.

[40] Avgerinos K. I., Spyrou N., Mantzoros C. S., Dalamaga M. (2019). Obesity and cancer risk: emerging biological mechanisms and perspectives. *Metabolism*.

[41] Marcello M. A., Cunha L. L., Batista F. A., Ward L. S. (2014). Obesity and thyroid cancer. *Endocrine-Related Cancer*.

[42] Fan Y. L., Li X. Q. (2015). Expression of leptin and its receptor in thyroid carcinoma: distinctive prognostic significance in different subtypes. *Clinical Endocrinology*.

[43] Dietlein M., Kahaly G., Kobe C., Derwahl K. M., Dietlein M. (2008). Obesity, energy regulation and thyroid function: is borderline elevated TSH-level the cause or secondary phenomenon of obesity. *Nuklearmedizin*.

[44] Li C., Zhou L., Dionigi G., Li F., Zhao Y., Sun H. (2020). The association between tumor tissue calcification, obesity, and thyroid cancer invasiveness in a cohort study. *Endocrine Practice*.

[45] Castagna M. G., Pinchera A., Marsili A. (2005). Influence of human body composition on serum peak thyrotropin (TSH) after recombinant human TSH administration in patients with differentiated thyroid carcinoma. *Journal of Clinical Endocrinology and Metabolism*.

[46] Barnabei A., Strigari L., Persichetti A. (2018). Indirect basal metabolism estimation in tailoring recombinant human TSH administration in patients affected by differentiated thyroid cancer: a hypothesis-generating study. *Frontiers in Endocrinology*.

[47] Kwon H., Han K. D., Park C. Y. (2019). Weight change is significantly associated with risk of thyroid cancer: a nationwide population-based cohort study. *Scientific Reports*.

[48] Youssef M. R., Reisner A. S. C., Attia A. S. (2021). Obesity and the prevention of thyroid cancer: impact of body mass index and weight change on developing thyroid cancer-pooled results of 24 million cohorts. *Oral Oncology*.

